# Extent of Follow-Up on Abnormal Cancer Screening in Multiple California Public Hospital Systems: A Retrospective Review

**DOI:** 10.1007/s11606-022-07657-4

**Published:** 2022-05-31

**Authors:** Elaine C. Khoong, Natalie A. Rivadeneira, Lucia Pacca, Dean Schillinger, David Lown, Palav Babaria, Neha Gupta, Rajiv Pramanik, Helen Tran, Tyler Whitezell, Ma Somsouk, Urmimala Sarkar

**Affiliations:** 1 Division of General Internal Medicine at Zuckerberg San Francisco General Hospital, Department of Medicine, University of California San Francisco, San Francisco, CA USA; 2 UCSF Center for Vulnerable Populations at Zuckerberg San Francisco General Hospital, San Francisco, CA USA; 3 California Health Care Safety Net Institute, Oakland, CA USA; 4 Alameda Health System, Oakland, CA USA; 5 Office of Informatics & Technology and Department of Emergency Medicine, Contra Costa Health Services, Martinez, CA USA; 6 Department of Family Medicine, Charles R. Drew University College of Medicine, Los Angeles, CA USA; 7 Department of Health Services at Los Angeles County, Los Angeles, CA USA; 8 Kern Medical, Bakersfield, CA USA; 9 Division of Gastroenterology, Department of Medicine, UCSF, San Francisco, CA USA

**Keywords:** safety-net system, cancer screening, colon cancer, breast cancer, cancer disparities

## Abstract

**Background:**

Inequitable follow-up of abnormal cancer screening tests may contribute to racial/ethnic disparities in colon and breast cancer outcomes. However, few multi-site studies have examined follow-up of abnormal cancer screening tests and it is unknown if racial/ethnic disparities exist.

**Objective:**

This report describes patterns of performance on follow-up of abnormal colon and breast cancer screening tests and explores the extent to which racial/ethnic disparities exist in public hospital systems.

**Design:**

We conducted a retrospective cohort study using data from five California public hospital systems. We used multivariable robust Poisson regression analyses to examine whether patient-level factors or site predicted receipt of follow-up test.

**Main Measures:**

Using data from five public hospital systems between July 2015 and June 2017, we assessed follow-up of two screening results: (1) colonoscopy after positive fecal immunochemical tests (FIT) and (2) tissue biopsy within 21 days after a BIRADS 4/5 mammogram.

**Key Results:**

Of 4132 abnormal FITs, 1736 (42%) received a follow-up colonoscopy. Older age, Medicaid insurance, lack of insurance, English language, and site were negatively associated with follow-up colonoscopy, while Hispanic ethnicity and Asian race were positively associated with follow-up colonoscopy. Of 1702 BIRADS 4/5 mammograms, 1082 (64%) received a timely biopsy; only site was associated with timely follow-up biopsy.

**Conclusion:**

Despite the vulnerabilities of public-hospital-system patients, follow-up of abnormal cancer screening tests occurs at rates similar to that of patients in other healthcare settings, with colon cancer screening test follow-up occurring at lower rates than follow-up of breast cancer screening tests. Site-level factors have larger, more consistent impact on follow-up rates than patient sociodemographic traits. Resources are needed to identify health system–level factors, such as test follow-up processes or data infrastructure, that improve abnormal cancer screening test follow-up so that effective health system–level interventions can be evaluated and disseminated.

**Supplementary Information:**

The online version contains supplementary material available at 10.1007/s11606-022-07657-4.

## BACKGROUND

Disparities exist in breast and colon cancer screening and treatment by race/ethnicity, socioeconomic status, and health insurance status.^1–6^ While cancer screening is influenced by insurance status and primary care access, additional disparities may emerge because of delays in the time between cancer screening, diagnosis, and treatment. Abnormal screening tests often require follow-up tests to confirm the diagnosis before definitive treatment. Little is known about the prevalence of poor follow-up in safety-net health systems, which are often under-resourced and disproportionately care for underserved communities. It is also unknown how much failures to follow-up abnormal screening tests contribute to cancer disparities in these settings.

Fecal immunochemical tests (FITs) are commonly used for colorectal cancer screening. For this screening approach to be successful, abnormal results require a timely follow-up colonoscopy for diagnostic evaluation. Mammography similarly screens for possible breast cancer; diagnosis can require follow-up studies after an abnormal screening mammogram, such as biopsy of a suspicious lesion.

Although there have been single- and multi-site studies on follow-up rates for abnormal FITs and/or mammograms,^7–11^ it has been difficult to capture the extent of follow-up of abnormal screening tests at a population level, particularly in safety-net systems. Unlike cancer diagnosis and treatment for which national databases exist, health systems have not been required to report data on follow-up of abnormal FITs or mammograms.

With electronic health records (EHR) adoption in many safety-net systems,^12,13^ it is now possible to harness electronically available quality measures. To leverage this opportunity, the California Medicaid program (i.e., Medi-Cal) implemented a pay for performance program known as the Public Hospital Redesign and Incentives in Medi-Cal program, or PRIME Program. PRIME incentivized public hospital systems to report and improve on their performance across several quality measures, including measures related to follow-up of abnormal FITs and mammograms.^14^

Concurrently, PRIME incentivized health systems to begin uniformly capturing race/ethnicity and language data from all patients. Therefore, this program provided a unique opportunity to investigate patient-level predictors of disparities in follow-up of abnormal FITs and mammograms. This report aims to describe patterns of performance in five California public hospital systems on follow-up of abnormal colon and breast cancer screening tests and explores the extent to which racial/ethnic disparities exist in these systems.

## METHODS

### Setting and Data Collection

Five California public hospital systems extracted data from EHRs from July 2015 to June 2017 about patient sociodemographic traits and clinical data. Within California, many public hospital systems are integrated with or closely connected to nearby community health centers and federally qualified health centers; therefore, the public hospital systems include both ambulatory and inpatient settings. Sites were asked to provide patient-level data using the same queries used for the PRIME metrics in the first 2 years of the PRIME program (year 1: July 1, 2015–June 30, 2016, year 2: July 1, 2016–June 30, 2017). The PRIME program consisted of required and optional metrics (though systems were required to choose some of the optional metrics). The metrics associated with follow-up of cancer screening tests were optional; therefore, for sites that did not report these metrics for the PRIME program, the study investigators sent the California Department of Health Care Services documentation on the metric specifications to each site’s staff. After receiving data, our study staff standardized reporting of race/ethnicity, language, and insurance type/coverage. Characteristics about sites and their processes for test follow-up were collected from publicly available data and elicited via email from site leaders. (See Appendix Table 1) The UCSF Institutional Review Board approved this study (15-18136).

### Outcome Variables

Among patients 50–74 years old, we measured two dichotomous outcomes related to colorectal and breast cancer follow-up for each year of the PRIME program: (1) failure to receive a colonoscopy in the same PRIME measurement year after an abnormal FIT test from the first six months of each year and (2) failure to receive a tissue biopsy within 21 days in the same PRIME measurement year after a mammogram rated as BIRADS 4 or 5 (i.e., a suspicious or highly suspicious mammogram) acquired at any point during that measurement year. Only abnormal FITs in the first 6 months of each measurement year were included to allow sufficient time for follow-up colonoscopy in the same measurement year. We chose these follow-up time points based on clinical practice guidelines and studies demonstrating adverse outcomes with further delays in diagnosis.^15–19^ We only included patients ≥ 50 years because at the time of this study, breast and colon cancer screening were recommended for all such patients by the US Preventive Services Task Force.^20,21^ For the mammogram-related outcome, we only included female patients.

### Predictor Variables

In addition to site (sites A–E), we included five patient-level sociodemographic characteristics: age, gender, race/ethnicity, preferred language, and insurance coverage/type. Age included four groups: 50–54, 55–59, 60–64, and 65+ years old. Preferred language was categorized into English, Spanish, and other language. Race/ethnicity was defined with five categories: non-Hispanic White, non-Hispanic Black/African American, Hispanic/Latinx (all races), non-Hispanic Asian, and other. Insurance was defined with five categories: Medicaid (including dual eligible beneficiaries), Medicare, private, uninsured, and other coverage (e.g., local health access programs).

### Statistical Analysis

We conducted robust Poisson regression^22^ to estimate adjusted relative risks of failing to receive follow-up and included all predictor variables (age [reference: 50–54 years], gender [reference: male], race/ethnicity [reference: non-Hispanic White], language [reference: English], insurance coverage/type [reference: private insurance], and site [reference: site A]) as fixed effects. Separate models were run for each metric. The model for FIT follow-up was run on the full dataset. The breast cancer model was run on imputed datasets because of missing sociodemographic data. Ten datasets were imputed and analyzed using SAS 9.4 proc mi procedure. We also conducted two sensitivity analyses. The first focused on addressing data quality concerns, which created models that removed data from sites with statistically significant changes in performance between years (which may have suggested EHR–extracted data was lower quality).^23^ We also performed sensitivity analyses to explore whether the timing of the follow-up biopsy of BIRADS 4/5 mammograms (e.g., 30 days, 60 days) impacted the findings.

## KEY RESULTS

### Included Participants

Our analyses included 4132 participants with an abnormal FIT and 1702 participants with a suspicious/highly suspicious mammogram (BIRADS 4/5) (Table 1). There was significant racial/ethnic and linguistic diversity in the study. Over 80% of participants across both measures identified as persons of color, and approximately half preferred a non-English language. Nearly 80% were publicly insured with Medicare and Medicaid, with a majority insured by Medicaid.

**Table 1:** Sociodemographic Characteristics of Included Participants

Trait	Abnormal FIT (*N* = 4132) *n* (%)	Suspicious/highly suspicious mammogram (BIRADS 4/5) (*N* = 1702) *n* (%)
Age (median)	60	59
Gender		
Male	1942 (47)	–
Female	2190 (53)	1702 (100)
Race/ethnicity		
Non-Hispanic White	717 (18)	302 (18)
Black/African American	704 (17)	246 (14)
Hispanic	1799 (44)	688 (40)
Asian	603 (15)	316 (19)
Other	279 (7)	125 (7)
Preferred language		
English	2228 (54)	802 (47)
Spanish	1411 (34)	652 (38)
Other	493 (12)	248 (15)
Insurance		
Private	284 (7)	158 (9)
Medicaid	2731 (66)	999 (59)
Medicare	750 (18)	259 (15)
Other	203 (5)	141 (8)
Uninsured	142 (4)	41 (2)
Site		
Site A	563 (14)	462 (27)
Site B	333 (8)	98 (6)
Site C	558 (14)	140 (8)
Site D	20 (1)	12 (1)
Site E	2658 (64)	990 (58)

30 (0.7%) missing race/ethnicity for abnormal FIT, 25 (1%) missing race/ethnicity, 104 (6%) missing insurance data for suspicious/highly suspicious abnormal mammogram

*BIRADS* Breast Imaging Reporting and Database System, *FIT* fecal immunochemical test

### Test Follow-Up Processes

There was variation in site processes for following up abnormal tests (summary in Table 2; details in appendix Table 1). Across all sites, abnormal FIT follow-up involved communication between the primary care provider (PCP), gastroenterology (GI) team, and the patient. Site A completed colonoscopies primarily internally while other systems used internal and external gastroenterologists. Most site processes involved multiple handoffs between the PCP, GI team, and a quality improvement team, but at site C, the GI care coordinator was primarily responsible for shepherding a patient through the entire process. There were similar patterns for mammogram follow-up but fewer handoffs between involved parties (PCPs and breast cancer/radiology). Most sites also primarily used internal services for follow-up biopsy after suspicious/highly suspicious mammograms.

**Table 2:** Health System Processes for Follow-Up of Abnormal FIT and BIRADS 4/5 Mammogram

Workflow step	Site A	Site B	Site C	Site D	Site E
Process for abnormal FIT follow-up					
Receives notification about FIT result	PCP (primary care provider)	PCP	PCP GI care coordinator	PCP	PCP
Notifies patient about FIT result	PCP	PCP	PCP	PCP	PCP
Places referral to colonoscopy	PCP	PCP	PCP	PCP	PCP
Location of colonoscopy	Internal	Internal and external	Internal and external	Internal and external	Internal and external
Schedules colonoscopy	GI team (if able to reach patient); if unable to reach patient, notify PCP	GI team	GI care coordinator with GI procedure scheduler	Schedulers (if reach patient); if unable to reach patient, notify PCP	GI team
Contacts patient about colonoscopy	GI team	GI team	GI care coordinator	Diagnostic Treatment Center staff	GI team
Orders bowel preparation	GI team	GI team	GI care coordinator	GI team	GI team
Educates patient about colonoscopy/bowel preparation	GI team via mailing; PCP	GI team via in-person class	GI care coordinator	Preop RN, part of GI team	GI team
Follow-up on unscheduled or unattended colonoscopies	PCP; QI team notifies clinics who notify PCP	PCP; cancer navigator contacts PCP	GI care coordinator will ask PCP to order	QI team contacts patient	GI team if able to reach patient; if not, notify PCP
Process for BIRADS 4/5 follow-up					
Receives notification about BIRADS 4/5 result	PCP; patient	PCP	PCP	PCP	PCP
Notifies patient about result and need for tissue sampling	Breast center; PCP	Radiology	Breast health nurse	PCP	Radiology
Orders tissue sampling	PCP (pre-ordered with mammogram order)	Radiology	PCP	PCP	PCP
Location of procedure	Internal	Internal and external	Internal	Internal	Internal
Schedules procedure	Breast center	Radiology	Breast health nurse	Procedure scheduler	Internal
Informs patient of appointment	Breast center	Radiology	Breast health nurse	Procedure nurse	Radiology
Follow-up on unscheduled or unattended procedures	Breast center; PCP; some clinics have breast cancer navigator	Radiology	Breast health nurse	Breast clinic team contacts patient	PCP; primary care nurse manager

Internal vs external location refers to whether the procedure is done within the same health system (and frequently on the same electronic health record) or referred to external specialists

*BIRADS* Breast Imaging Reporting and Database System, *FIT* fecal immunochemical test, *GI* gastroenterology, *PCP* primary care provider, *QI* quality improvement

### Follow-Up of Abnormal FIT

**Fig. 1:**
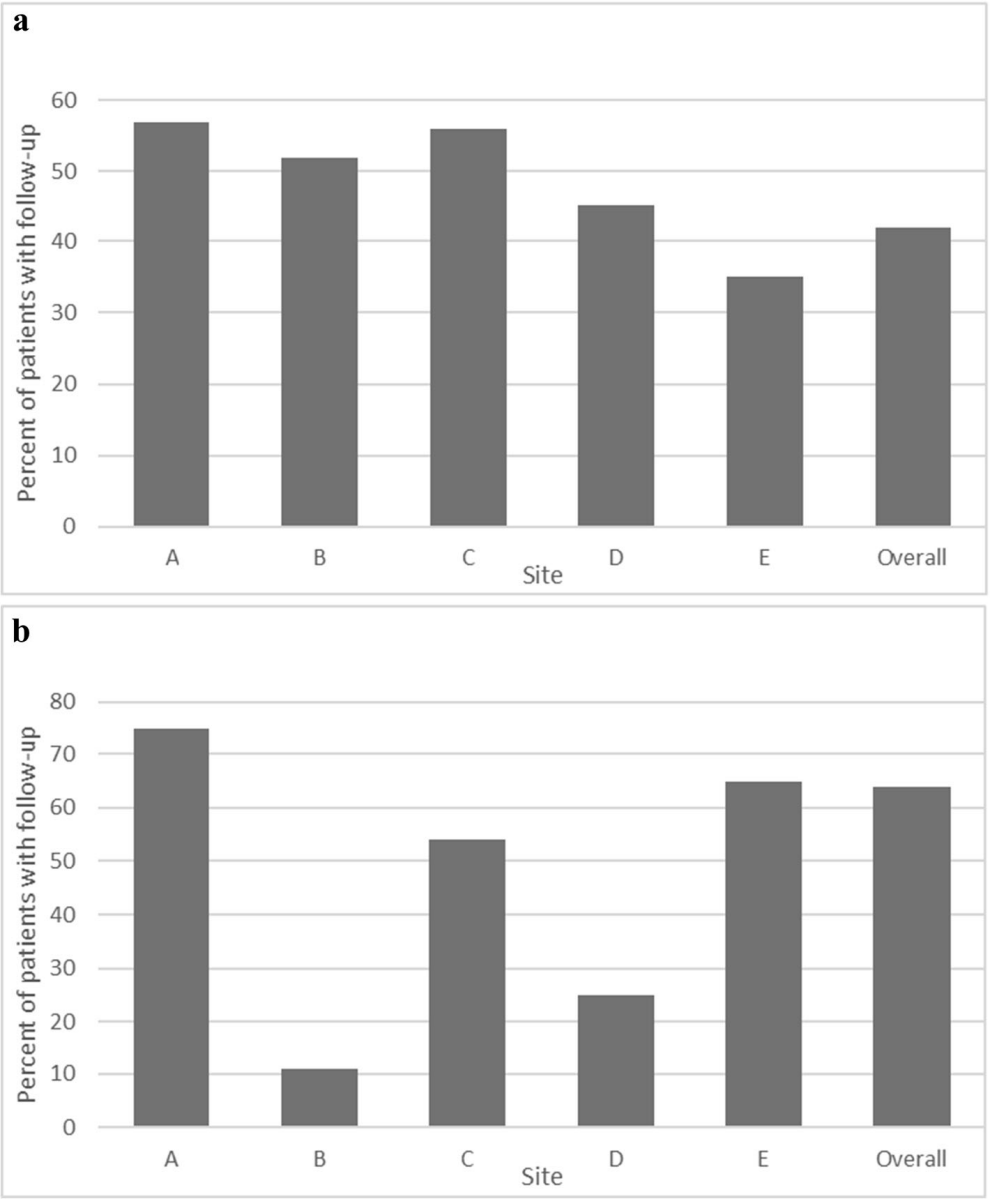
**a** Rates of follow-up colonoscopy after abnormal FIT. **b** Rates of timely follow-up biopsy (21 days) after a highly suspicious mammogram (BIRADS 4/5).

**Fig. 2:**
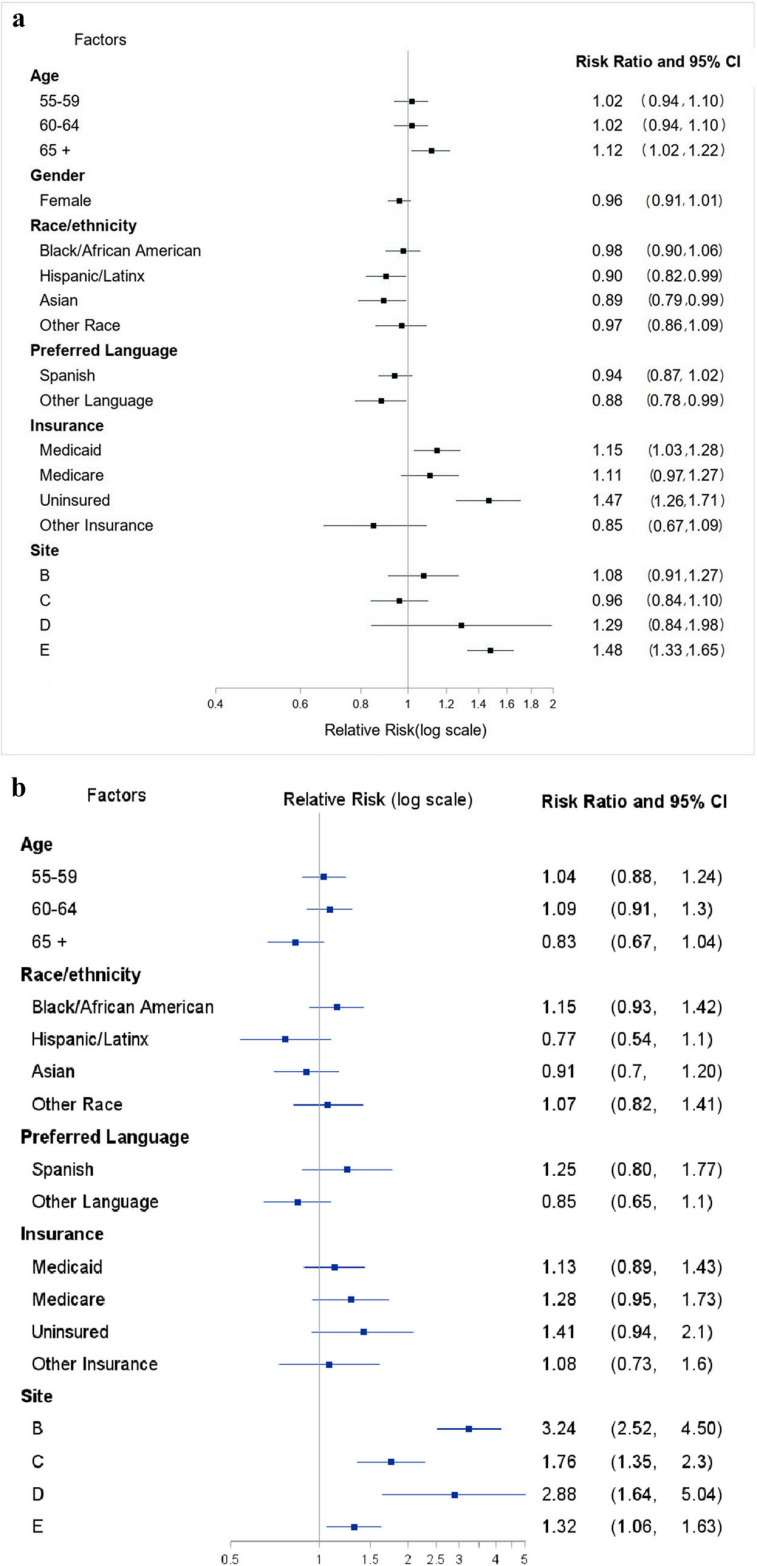
**a** Risk of failing to receive colonoscopy after abnormal FIT. Reference categories are age (50–54 yo), gender (male), race/ethnicity (White), preferred language (English), insurance (private), and site (A). This figure presents results of the adjusted model that controlled for age, gender, race/ethnicity, preferred language, insurance, and site. **b** Risk of failing to receive a timely biopsy (within 21 days) after a suspicious/highly suspicious mammogram (BIRADS 4/5). Reference categories are age (50–54 yo), race/ethnicity (White), preferred language (English), insurance (private), and site (A). This figure presents results of the adjusted model that controlled for age, race/ethnicity, preferred language, insurance, and site.

Among the 4132 individuals who had an abnormal FIT, 1736 (42%) completed a follow-up colonoscopy within the same year of the PRIME program (Fig. 1a). In adjusted analyses, age, race/ethnicity, language, insurance type/status, and site were significantly associated with follow-up (Fig. 2a; Appendix Table 2 also contains unadjusted analyses). Individuals ≥ 65 years old (aRR = 1.12; 95% CI: [1.02, 1.22]) vs 51–54 years old as well as uninsured individuals (aRR = 1.47; 95% CI: [1.26, 1.71]) and individuals insured by Medicaid (aRR = 1.15; 95% CI: [1.03, 1.28]) relative to privately insured patients had higher risk of failing to receive a follow-up colonoscopy. Individuals that spoke other languages (not English or Spanish) relative to English speakers (aRR = 0.88; 95% CI: [0.78, 0.99]) as well as individuals that identified as Asian (aRR = 0.89; 95% CI: [0.79, 0.99]) or Hispanic (aRR = 0.90; 95% CI: [0.82, 0.99]) relative to non-Hispanic White individuals had lower risk of failing to receive a follow-up colonoscopy. Patients at site E (aRR = 1.48; 95% CI: [1.33, 1.65]) had higher risk of failing to receive follow-up than patients at site A.

### Follow-Up of Suspicious/Highly Suspicious Mammogram (BIRADS 4/5)

Among 1702 women with a BIRADS 4/5 mammogram, 1082 (64%) received a biopsy within 21 days (Fig. 1b). While the unadjusted analysis showed several significant predictors (Appendix Table 3), in the multivariable analysis, only site was significantly associated with follow-up; there were no significant patient-level predictors. In comparison to site A participants, participants at all other sites had higher risk of failing to receive a follow-up biopsy (Fig. 2b; Appendix Table 3).

Findings, in particular the importance of site, were generally unchanged and robust to sensitivity analyses when sites with variability in performance were excluded or when other definitions of a “timely” biopsy (e.g., 30 days, 60 days) after a suspicious or highly suspicious mammogram were used (Appendix Tables 4 and 5).

## DISCUSSION

Our study, which relied on site-wide, EHR data derived from safety-net systems, is consistent with prior studies in various clinical settings showing inconsistency and variability in receipt of the follow-up necessary to pursue definitive cancer diagnosis after abnormal screening tests.^8,11,23,24^ It is notable that despite caring for a patient population with increased vulnerabilities, these public hospital systems achieved similar follow-up rates to prior studies that have shown 40–60% of individuals receive a colonoscopy after an abnormal FIT while 50–70% receive follow-up after incomplete/suspicious mammograms.^11,23–28^ Based on prior literature, we anticipated that patient-level characteristics would largely explain such variation. However, among these five public health systems, we observed that site-level factors were more important contributors to differences in follow-up rates. After adjusting for site and other sociodemographic factors, the only racial/ethnic disparities we identified were lower risk of failure to receive follow-up colonoscopy among Hispanic and Asian individuals and the individuals with a preferred language other than English or Spanish.

Our findings suggest that at the current level of performance, health system factors, such as personnel, test follow-up processes, and data infrastructure (e.g., disease registries), significantly impact follow-up of abnormal cancer screening results. The importance of considering multi-level barriers at the patient and system levels has been demonstrated in prior studies.^29–32^ Each health system had different workflows to follow-up each test result. In some cases, these workflows were not standardized within the health system. Importantly, all these test results required coordination between multiple departments and sometimes external health systems. These complicated processes may result in “dropped balls.” Monitoring and follow-up of sub-critical tests (i.e., test results that need follow-up in weeks to months to years) is a well-known gap in ambulatory patient safety but has been less studied in cancer screening and diagnosis.^33,34^ While our study was not powered or designed to discern specific system-level factors that influence follow-up, we observed a pattern that health systems using processes involving coordination with fewer individuals or requiring fewer handoffs in responsibility (e.g., internal referral within a system; clearly identified individual or small group to ensure follow-up) appeared to have a lower risk of failing to provide appropriate follow-up. For example, site C assigned a single care coordinator to ensure follow-up of all abnormal FIT results and site A used only internal colonoscopy referrals; these two sites had better performance compared to site E, which generated both internal and external referrals coordinated between the PCP and gastroenterology team, without a clearly identified responsible party within either the gastroenterology or primary care team. Attention to these processes through development of standardized workflows has been shown to improve follow-up.^29^

An additional finding is that we observed no consistency in performance within health systems across these two outcomes. Workflows to follow-up an abnormal FIT versus an abnormal mammogram not only are different but also require coordination across different groups. One site may have a high-quality process to follow-up abnormal FITs, but having such a process did not translate to quality processes for following up a suspicious mammogram. For example, site A had similar performance to sites B, C, and D on abnormal FIT follow-up but was significantly worse than all other sites for abnormal mammogram follow-up. Even within systems, there may be variation in performance; larger systems with more clinicians in complex networks have greater difficulty standardizing practice change or implementing innovations to improve care.^35^

While no patient-level contributors were significantly associated with follow-up of a suspicious/highly suspicious mammogram, we did find that older age, having no insurance, and being insured by Medicaid were associated with higher risk of failing to receive colonoscopy after an abnormal FIT. Our findings are consistent with prior studies that showed older patients are less likely to receive a follow-up colonoscopy after an abnormal FIT.^11,30,31^ Some studies have found that patient behaviors (refusal) or physician behaviors (inappropriate screening) may explain some of these differences in follow-up rates by age.^31^ Insurance status/type are markers for other social needs (such as housing or transportation),^36^ which may be particularly important for colonoscopy completion. Colonoscopies require patients to complete a bowel preparation, have reliable access to a bathroom during the bowel preparation, and have transportation after the procedure. Social risk factors, such as unreliable transportation access, housing insecurity, or limited health literacy may undermine a successful colonoscopy.^11^

We also found that Hispanic ethnicity, Asian race, and a non-English/Spanish language preference were associated with a lower risk of failing to receive a follow-up colonoscopy. While some studies found populations of color are less likely to receive a follow-up colonoscopy after an abnormal FIT,^30^ studies conducted within systems where insurance and access may be similar (e.g., Veterans Health Affairs or safety-net systems) have found racial/ethnic minorities are more likely than non-Hispanic White populations to acquire a colonoscopy after an abnormal FIT.^7,31^ While few studies have specifically assessed the impact of language preference on abnormal FIT follow-up, our team has shown patients with non-English language preference are more likely to adhere to follow-up recommendations.^7^ The relationship between race/ethnicity, language, and adherence to follow-up colonoscopy warrants further exploration in even more diverse patient populations, as cultural differences not fully captured by self-identified race/ethnicity and language preference may be at play, such as differences associated with immigration status.^37^

This study has several limitations. We could not account for all differences in patient-level factors, such as unmet social needs. Prior research on FIT follow-up revealed many patient-level social needs are barriers to obtaining colonoscopy.^7,38,39^ We also could not assess if failures in follow-up are due to patient, clinician, or health system actions. Since we only have data from the first 2 years of the PRIME program, we have limited capacity to determine whether this pay-for-performance program impacted quality of care. We only included abnormal FIT data from the first 6 months of each measurement year; however, prior studies have shown nearly 85% of colonoscopies are acquired within the first 6 months after an abnormal FIT, and outcomes worsen only if colonoscopies are acquired more than 12 months after the abnormal FIT.^15,16,40^ Moreover, the follow-up rates identified in our study were consistent with prior literature. Each site oversaw their own data validation procedures, which may have introduced variations in data quality, but our sensitivity analyses confirmed this had limited effect on key results. There is potential that we may have had incomplete data capture if tests were acquired outside the hospital system. We only studied five public hospital systems and their integrated ambulatory settings in California; these integrated systems may be less common in other states, which may limit generalizability to other Medicaid populations and providers. However, these five systems collectively care for nearly 500,000 Medicaid patients and provide over 3 million outpatient visits annually across California. This study is strengthened by our inclusion of a diverse sample and health system–level measurement of outcomes that are understudied.

Despite these limitations, we believe this study has several implications. Researchers, clinicians, and patient advocates should further explore how failures to follow-up abnormal screening tests may contribute to worse outcomes in cancer diagnosis and care. In our sample, we found system-level variation to be a more important predictor than sociodemographic characteristics. Therefore, resources should be devoted to understanding the factors underlying health system variation that affect whether patients receive appropriate follow-up of abnormal screening tests, such as information technology infrastructure, test follow-up workflows, or provision of patient support. In this study, high performance in one area did not translate to high performance in another area, suggesting variation in specific test workflows (rather than health-system-specific traits) is important. To facilitate better test follow-up, tools such as root cause analysis or process mapping may provide greater understanding of why a specific health system performs well on a specific abnormal test; findings from these investigations may suggest additional resources, such as staff or infrastructure, are needed to address vulnerabilities in the follow-up process. Payors should consider how to provide health systems with the resources to adequately support patient safety improvements. After identifying root causes, researchers and practitioners should systematically assess whether specific interventions—such as reducing handoffs among different departments or identifying a small group of individuals to have primary responsibility for ensuring follow-up—increase follow-up. If these practices are effective, they can be documented, disseminated, and tested in other settings to improve outcomes.

## CONCLUSION

Consistent with other studies and settings, within these public hospital systems, failure to follow-up on abnormal colon and breast cancer screening tests is frequent and may contribute to worse cancer outcomes. If healthcare systems continue to focus only on increasing cancer screening rates without attention to whether patients receive follow-up for abnormal screening tests, systems will continue to fail to achieve optimal cancer outcomes. Health system factors appear to affect follow-up of abnormal cancer screening tests; quality improvement experts and implementation scientists should investigate why specific health systems may perform well on following up a specific test. Additional examination of previously understudied patient-level factors, such as transportation or housing, may deepen our understanding of these gaps in cancer care and inform future attempts to improve follow-up of abnormal cancer screening tests.

## Supplementary Information

ESM 1 (DOCX 45 kb)

## Data Availability

All data generated or analyzed during this study are included in this published article and its supplementary information files.
