# Supplementary material for: Extent of Follow-Up on Abnormal Cancer Screening in Multiple California Public Hospital Systems: A Retrospective Review
Source: J Gen Intern Med. 2022 May 31;38(1):21–9. doi: 10.1007/s11606-022-07657-4 (PMC9849534; doi:10.1007/s11606-022-07657-4)
Supplement: Supplementary file 1 — (DOCX 45 kb) [file 11606_2022_7657_MOESM1_ESM.docx]

**APPENDICES**

**Appendix Table 1**: Health System Characteristics During Study Years (06/2015 – 07/2017)

|  | Site A | Site B | Site C | Site D | Site E |
| --- | --- | --- | --- | --- | --- |
| Outpatient Visits | 550K-650K | 400K -500K | 450K -550K | 100K -200K | 1200K -1300K |
| PRIME Population | 50K-60K | 45K-55K | 85K-95K | 25K-35K | 255K-265K |
| Race/ethnicity of patient population | 22% Asian  15% Black  28% Hispanic  1% Native American | 16% Asian / Pacific Islander  27% Black  43% Hispanic  0% Other | 14% Asian  16% Black  37.5% Hispanic  1.3% American Indian / Alaskan Native & Native Hawaiian / Pacific Islander | 2% Asian  14% Black  58% Hispanic  7% Native American | 2% Asian  25% Black  50% Hispanic  10% Native Hawaiian / Pacific Islander |
| Age of patient population | 12% 0-18  39% 18-44  32% 45-64  17% 65+ | 12% 0-15  9% 16-24  71% 25-64  8% 65+ | 28% 0-17  42% 18-49  24% 50-69  7% 75+ | 26% 0-20  58% 21-50  24% 51-70  5% 71+ | 8% 0-18  25% 19-44  68% 45-74  3% 75+ |
| Insurance coverage of patient population | 1% Commercial  57% Medicaid  21% Medicare  11% Other  10% Uninsured | 10% Commercial  48% Medicaid  19% Medicare  4% Other  19% Uninsured | 2% Commercial  85% Medicaid  9% Medicare  1% Other  1% Uninsured | 10% Commercial  68% Medicaid  8% Medicare  10% Other  3% Uninsured | 3% Commercial  68% Medicaid  8% Medicare  11% Other  10% Uninsured |
| Electronic health record ^a^ | Not enterprise | Not enterprise | Enterprise | Not enterprise | Enterprise |
| Major operational undertakings in 2015 – 2017 time period | - Expanded electronic consultation services  - Moved radiology imaging and archiving (PACS) into new system  - Adopted Lean Improvement thinking | - Implemented ambulatory electronic health record (NextGen)  - Kaizen to improve radiology processes  - Hired of cancer navigators to track and perform outreach to patients with abnormal mammograms | - Implemented electronic consultation for specialty care  - Systemwide quality improvement trainings which significantly changed system culture  - Implementation of wellbeing screen, which combined Staying Healthy Assessment with substance use screening, brief intervention, and referral tool | - Implemented i2i disease registry  - Hired staff for outpatient performance improvement and data analysis  - Monthly meetings with clinical leaders to discuss PRIME goals and action plans. | - Implemented care management registry in electronic health record |

| Process for Abnormal FIT Follow-Up | | | | | |
| --- | --- | --- | --- | --- | --- |
| Follow-up on abnormal FIT and orders colonoscopy (internally or externally) | PCP (primary care provider) receives abnormal FIT result and placed colonoscopy order after communicating results to patient.  Colonoscopies done internally in same system | PCP receives result, orders colonoscopy, and communicates results to patients  Due to access issues, south county clinics refer to private practice GI doctors but otherwise for most part done internally in health system | GI care coordinator follows all abnormal FIT results. PCP or ordering clinician expected to notify patient about abnormal FIT results and order colonoscopy but GI care coordinator will also follow-up if missed by PCP / ordering clinician.  Colonoscopies are performed internally and externally. A clinician triages the referral and decides if colonoscopy should be referred. | The PCP follows up on the abnormal FIT results and places the colonoscopy referral.  Colonoscopies are performed both primarily internally, but will schedule externally if patient has an established relationship with external provider | PCP reviews the abnormal FIT results and places colonoscopy referral.  Colonoscopies can be completed either internally or externally depending on the clinic site. |
| Scheduling colonoscopy, ordering bowel prep, and notifying patient | After consult placed for colonoscopy, GI team contacts patient to discuss bowel regimen and schedule colonoscopy  If patient cannot be contacted, then no colonoscopy is scheduled and the PCP / ordering clinician notified.  GI prescribes bowel prep / laxative. | At time of study, patients required to attend a class about preparing for a colonoscopy before colonoscopy scheduled.  Colonoscopy scheduled by GI lab.  GI prescribes bowel prep / laxative. | The GI care coordinator works with the GI procedure scheduler to schedule the colonoscopy.  The GI procedures scheduler ensures the bowel prep / laxative is prescribed and helps navigate patient to the appointment. | Schedulers contact the patient to schedule colonoscopy. If unable to reach patient, a communication note is left in the patient’s chart notifying the ordering clinician that the patient was not reached.  Once the colonoscopy is scheduled, the staff of the Diagnostic Treatment Center communicates with the patient.    The pre procedure prep is discussed with patient by the GI Team RN 1 week before the colonoscopy. If a prescription is necessary, it will be called in to the patient’s pharmacy by the GI team. | This varies by site. In general, any bowel prep prescription is ordered by the GI team. Otherwise, there is variation in who schedules the colonoscopy and navigates the patient after a scheduled a colonoscopy |
| Population level review if patients are lost to follow-up or miss appointments | None. Each clinic has its own process. | Cancer navigators review list of patients with positive FIT without colonoscopy order and message PCP to order | The GI nurse manager will review population level metrics to address gaps in follow-up. | The population health team, alongside the QI team review population health metrics to address gaps in follow-up | Varies by site |

| Process for BIRADS 4/5 f/u | | | | | |
| --- | --- | --- | --- | --- | --- |
|  | When PCP orders mammogram, it is ordered with a pre-approval for biopsy if needed.  If the mammogram is read as BIRADS 4/5, then a tissue sample may be acquired same day. Otherwise, the breast imaging center will contact the patient with the abnormal result to schedule a time to acquire a tissue sample. These tests are all conducted internally. | Radiology places order for IR guided biopsy  Once authorization is obtained, patient is contacted and scheduled.    Most is done internally, but south county clinics often order mammograms outside system | Patients notified same way as for BIRADS 0.  When tissue sampling is recommended, breast health nurses contact the patient to schedule them with a breast health specialist to discuss the results. That clinician will order the biopsy.  Ultrasound guided biopsies are mostly done internally. Occasionally they will be done at outside facilities if the patient/clinician prefers. All stereotactic biopsies and MRI guided biopsies are performed externally.  Breast health nurses follow the BIRAD 4/5s and make sure that the biopsies are completed. | Same as process for BIRADS 0  PCP orders the biopsy. Once authorization is obtained, the breast clinic team contacts patient to schedule the procedure. | Same as process for BIRADS 0 |

^a^ An enterprise electronic health record (EHR) was defined as having the same, integrated EHR for inpatient care, ambulatory care, laboratory, imaging, and pathology results, and registry management.

**Appendix Table 2:** Abnormal FIT follow-up rates, unadjusted relative risk, and adjusted relative risk

| **Trait** | Follow-Up Rate: # of colonoscopy/ # abnormal FIT (%)  (N = 4132) | Unadjusted Relative Risk | 95% CI | | Adjusted Relative Risk | 95% CI | |
| --- | --- | --- | --- | --- | --- | --- | --- |
| **Age** | | | | | | | |
| 50-54 | 308/709 (43) | Reference | | | | | |
| 55-59 | 480/1149 (42) | 1.03 | 0.95 | 1.12 | 1.02 | 0.94 | 1.10 |
| 60-64 | 555/1320 (42) | 1.02 | 0.95 | 1.11 | 1.02 | 0.94 | 1.10 |
| 65 + | 393/954 (41) | 1.04 | 0.96 | 1.13 | 1.12* | 1.02 | 1.22 |
| **Gender** | | | | | | | |
| Male | 774/1942 (40) | Reference | | | | | |
| Female | 962/2190 (44) | 0.93* | 0.89 | 0.98 | 0.96 | 0.91 | 1.01 |
| **Race/ethnicity** | | | | | | | |
| White | 288/717 (40) | Reference | | | | | |
| Black/African American | 274/704 (39) | 1.02 | 0.94 | 1.11 | 0.98 | 0.90 | 1.06 |
| Hispanic/Latinx | 719/1799 (40) | 1.00 | 0.93 | 1.08 | 0.90* | 0.82 | 0.99 |
| Asian | 320/603 (53) | 0.78 * | 0.71 | 0.87 | 0.89* | 0.79 | 0.99 |
| Other race | 120/279 (43) | 0.95 | 0.85 | 1.07 | 0.97 | 0.86 | 1.09 |
| **Preferred Language** | | | | | | | |
| English | 892/2228 (40) | Reference | | | | | |
| Spanish | 567/1411 (40) | 1.00 | 0.94 | 1.05 | 0.94 | 0.87 | 1.02 |
| Other language | 277/493 (56) | 0.73* | 0.66 | 0.81 | 0.88* | 0.78 | 0.99 |
| **Insurance** | | | | | | | |
| Private | 128/284 (45) | Reference | | | | | |
| Medicaid | 1083/2731 (40) | 1.10 | 0.98 | 1.23 | 1.15* | 1.03 | 1.28 |
| Medicare | 338/750 (45) | 1.00 | 0.88 | 1.13 | 1.11 | 0.97 | 1.27 |
| Uninsured | 42/143 (29) | 1.29* | 1.11 | 1.49 | 1.47* | 1.26 | 1.71 |
| Other Insurance | 134/203 (66) | 0.62 | 0.50 | 0.77 | 0.85 | 0.67 | 1.09 |
| **Site** | | | | | | | |
| A | 323/563 (57) | Reference | | | | | |
| B | 174/333 (52) | 1.12 | 0.97 | 1.30 | 1.08 | 0.91 | 1.27 |
| C | 311/558 (56) | 1.04 | 0.91 | 1.19 | 0.96 | 0.84 | 1.10 |
| D | 9/20 (45) | 1.29 | 0.86 | 1.94 | 1.29 | 0.84 | 1.98 |
| E | 919/2658 (35) | 1.53* | 1.39 | 1.70 | 1.48* | 1.33 | 1.65 |

^*^ p < 0.05

**Appendix Table 3.** Suspicious or highly suspicious mammogram (BIRADS 4 or 5) follow-up rates, unadjusted relative risk, and adjusted relative risk

| **Trait** | Follow-Up Rate: # biopsies / # BIRADS 4/5 mammograms (%) (N = 1702) | Unadjusted Relative Risk | 95% CI | | Adjusted Relative Risk | 95% CI | |
| --- | --- | --- | --- | --- | --- | --- | --- |
| **Age** |  |  |  |  |  |  |  |
| 50-54 | 263/416 (63) | Reference | | | Reference | | |
| 55-59 | 292/470 (62) | 1.03 | 0.87 | 1.22 | 1.04 | 0.88 | 1.24 |
| 60-64 | 266/427 (62) | 1.03 | 0.86 | 1.22 | 1.09 | 0.91 | 1.30 |
| 65 + | 261/389 (67) | 0.89 | 0.74 | 1.08 | 0.83 | 0.67 | 1.04 |
| **Race/ethnicity** |  |  |  |  |  |  |  |
| White |  | Reference | | | Reference | | |
| Black/African American | 133/246 (54) | 1.18 | 0.96 | 1.44 | 1.15 | 0.93 | 1.42 |
| Hispanic/Latinx | 445/688 (65) | 0.92 | 0.77 | 1.09 | 0.77 | 0.54 | 1.10 |
| Asian | 224/316 (71) | 0.75* | 0.60 | 0.93 | 0.91 | 0.70 | 1.20 |
| Other race | 78/125 (62) | 0.97 | 0.74 | 1.27 | 1.07 | 0.82 | 1.41 |
| **Preferred Language** |  |  |  |  |  |  |  |
| English | 486/802 (61) | Reference | | | Reference | | |
| Spanish | 417/652 (64) | 0.91 | 0.80 | 1.05 | 1.25 | 0.80 | 1.77 |
| Other language | 179/248 (72.18) | 0.71* | 0.57 | 0.87 | 0.85 | 0.65 | 1.19 |
| **Insurance** |  |  |  |  |  |  |  |
| Private |  | Reference | | | Reference | | |
| Medicaid | 628/999 (63) | 1.14 | 0.91 | 1.44 | 1.13 | 0.89 | 1.43 |
| Medicare | 148/259 (57) | 1.30 | 0.99 | 1.69 | 1.28 | 0.95 | 1.73 |
| Uninsured | 17/41 (41) | 1.74* | 1.18 | 2.57 | 1.41 | 0.94 | 2.10 |
| Other Insurance | 105/141 (74) | 0.80 | 0.57 | 1.12 | 1.08 | 0.73 | 1.60 |
| **Site** |  |  |  |  |  |  |  |
| A | 345/462 (75) | Reference | | | Reference | | |
| B | 11/98 (11) | 3.51* | 2.81 | 4.37 | 3.24* | 2.52 | 4.50 |
| C | 76/140 (54) | 1.81* | 1.42 | 2.30 | 1.76* | 1.35 | 2.30 |
| D | 3/12 (25) | 2.96* | 1.72 | 5.09 | 2.88* | 1.64 | 5.04 |
| E | 647/990 (65) | 1.37* | 1.16 | 1.62 | 1.32* | 1.06 | 1.63 |

Note: 25 (1%) missing race/ethnicity, 104 (6%) missing insurance data for high-risk abnormal mammogram. *p < 0.05

**Appendix Table 4.** Sensitivity analyses (data quality)

| **Abnormal FIT Follow-up**  (removed site B and E) | | | | **Suspicious/highly suspicious mammogram (BIRADS 4/5) follow-up** (removed site D) | | | |
| --- | --- | --- | --- | --- | --- | --- | --- |
|  |  |  |  |  |  |  |  |
|  | **aRR** | **95% CI** | |  | **aRR** | **95% CI** | |
| **Age** |  |  |  | **Age** |  |  |  |
| 50-54 | Reference | | | 50-54 | Reference | | |
| 55-59 | 1.02 | 0.94 | 1.11 | 55-59 | 1.06 | 0.89 | 1.26 |
| 60-64 | 1.02 | 0.94 | 1.11 | 60-64 | 1.08 | 0.90 | 1.30 |
| 65 + | 1.12 | 1.03 | 1.23 | 65 + | 0.82 | 0.66 | 1.03 |
| **Gender** |  |  |  |  |  |  |  |
| Male | Reference | | |  |  |  |  |
| Female | 0.96 | 0.91 | 1.01 |  |  |  |  |
| **Race/ethnicity** | |  |  | **Race/ethnicity** | |  |  |
| White | Reference | | | White | Reference | | |
| Black / African American | 1.00 | 0.92 | 1.09 | Black/ African American | 1.14 | 0.92 | 1.41 |
| Hispanic/ Latinx | 0.96 | 0.87 | 1.05 | Hispanic/ Latinx | 0.72 | 0.50 | 1.06 |
| Asian | 0.87 | 0.77 | 0.98 | Asian | 0.90 | 0.69 | 1.16 |
| Other race | 0.97 | 0.86 | 1.09 | Other race | 1.06 | 0.80 | 1.40 |
| **Preferred Language** | |  |  | **Preferred Language** | |  |  |
| English |  |  |  | English | Reference | | |
| Spanish | 0.95 | 0.87 | 1.03 | Spanish | 1.33 | 0.92 | 1.91 |
| Other language | 0.82 | 0.72 | 0.94 | Other language | 0.86 | 0.66 | 1.11 |
| **Insurance** |  |  |  | **Insurance** |  |  |  |
| Private | Reference | | | Private | Reference | | |
| Medicaid | 1.10 | 0.99 | 1.23 | Medicaid | 1.13 | 0.89 | 1.43 |
| Medicare | 1.00 | 0.88 | 1.14 | Medicare | 1.33 | 0.98 | 1.80 |
| Uninsured | 1.38 | 1.19 | 1.61 | Uninsured | 1.41 | 0.94 | 2.11 |
| Other insurance* | 0.60 | 0.46 | 0.78 | Other insurance^*^ | 1.09 | 0.74 | 1.62 |
| **Site** |  |  |  | **Site** |  |  |  |
| A | Reference | | | A | Reference | | |
| B | - | - | - | B | 3.22 | 2.51 | 4.14 |
| C* | 0.71 | 0.64 | 0.79 | C | 1.76 | 1.35 | 2.31 |
| D* | 0.90 | 0.60 | 1.36 | D | - | - | - |
| E | - | - | - | E | 1.32 | 1.07 | 1.63 |

*^†^Estimate higher than expected*

** Estimate lower than expected*

**Appendix Table 5.** Sensitivity analyses (timing of follow-up biopsy)

| **Suspicious/highly suspicious mammogram (BIRADS 4/5) follow-up** (30 day follow-up) | | | | **Suspicious/highly suspicious mammogram (BIRADS 4/5) follow-up** (60 day follow-up) | | | | **Suspicious/highly suspicious mammogram (BIRADS 4/5) follow-up** (ever received follow-up) | | | |
| --- | --- | --- | --- | --- | --- | --- | --- | --- | --- | --- | --- |
|  |  |  |  |  |  |  |  |  |  |  |  |
|  | **aRR** | **95% CI** | |  | **aRR** | **95% CI** | |  | **aRR** | **95% CI** | |
| **Age** |  |  |  | **Age** |  |  |  | **Age** |  |  |  |
| 50-54 | Reference | | | 50-54 | Reference | | | 50-54 | Reference | | |
| 55-59 | 1.02 | 0.83 | 1.25 | 55-59 | 1.18 | 0.89 | 1.57 | 55-59 | 1.06 | 0.78 | 1.45 |
| 60-64 | 1.07 | 0.87 | 1.32 | 60-64 | 1.20 | 0.89 | 1.62 | 60-64 | 1.10 | 0.80 | 1.52 |
| 65 + | 0.70* | 0.54 | 0.91 | 65 +*^†^* | 1.07 | 0.75 | 1.51 | 65 + | 0.91 | 0.62 | 1.34 |
| **Race/ethnicity** | |  |  | **Race/ethnicity** | |  |  | **Race/ethnicity** | |  |  |
| White | Reference | | | White | Reference | | | White | Reference | | |
| Black / African American | 1.20 | 0.94 | 1.53 | Black/ African American | 1.20 | 0.88 | 1.65 | Black/ African American | 1.18 | 0.82 | 1.68 |
| Hispanic/ Latinx | 0.75 | 0.49 | 1.13 | Hispanic/ Latinx | 0.56 | 0.32 | 0.97 | Hispanic/ Latinx | 0.69 | 0.39 | 1.24 |
| Asian | 0.89 | 0.66 | 1.20 | Asian | 0.75 | 0.51 | 1.20 | Asian | 0.71 | 0.46 | 1.20 |
| Other race | 0.99 | 0.71 | 1.38 | Other race*** | 0.76 | 0.48 | 1.22 | Other race | 0.95 | 0.58 | 1.55 |
| **Preferred Language** | |  |  | **Preferred Language** | |  |  | **Preferred Language** | |  |  |
| English |  |  |  | English | Reference | | | English | Reference | | |
| Spanish | 1.21 | 0.80 | 1.81 | Spanish | 1.33 | 0.80 | 2.29 | Spanish | 1.15 | 0.80 | 2.02 |
| Other language | 0.88 | 0.65 | 1.19 | Other language | 0.95 | 0.64 | 1.43 | Other language | 1.12 | 0.72 | 1.74 |
| **Insurance** |  |  |  | **Insurance** |  |  |  | **Insurance** |  |  |  |
| Private | Reference | | | Private | Reference | | | Private | Reference | | |
| Medicaid | 1.08 | 0.82 | 1.43 | Medicaid | 1.18 | 0.79 | 1.76 | Medicaid | 1.21 | 0.77 | 1.92 |
| Medicare | 1.33 | 0.93 | 1.89 | Medicare | 1.15 | 0.71 | 1.88 | Medicare | 1.28 | 0.74 | 2.21 |
| Uninsured | 1.40 | 0.88 | 2.25 | Uninsured | 1.47 | 0.76 | 2.84 | Uninsured | 1.84 | 0.91 | 3.69 |
| Other insurance | 1.02 | 0.65 | 1.59 | Other insurance^*^ | 1.12 | 0.62 | 2.01 | Other insurance | 1.23 | 0.65 | 2.32 |
| **Site** |  |  |  | **Site** |  |  |  | **Site** |  |  |  |
| A | Reference | | | A | Reference | | | A | Reference | | |
| B | 3.41* | 2.59 | 4.50 | B | 3.50 | 2.48 | 4.50 | B | 3.07 | 2.11 | 4.50 |
| C | 1.72* | 1.27 | 2.32 | C | 1.73 | 1.19 | 2.52 | C | 1.67 | 1.12 | 2.51 |
| D | 3.23* | 1.77 | 5.87 | D | 4.01 | 2.00 | 8.04 | D | 3.24 | 1.45 | 7.23 |
| E | 1.08 | 0.84 | 1.37 | E*** | 0.71 | 0.52 | 0.98 | E* | 0.62 | 0.44 | 0.88 |

*^†^Estimate higher than expected*

** Estimate lower than expected*
